# Experimentation in 5G and beyond Networks: State of the Art and the Way Forward

**DOI:** 10.3390/s23249671

**Published:** 2023-12-07

**Authors:** Giuseppe Caso, Özgü Alay, Anna Brunstrom, Harilaos Koumaras, Almudena Díaz Zayas, Valerio Frascolla

**Affiliations:** 1 Department of Mathematics and Computer Science, Karlstad University, 65188 Karlstad, Sweden; ozgua@ifi.uio.no (Ö.A.); anna.brunstrom@kau.se (A.B.); 2 Department of Informatics, University of Oslo, 0373 Oslo, Norway; 3 NCSR “Demokritos”, Institute of Informatics and Telecommunications, 15341 Agia Paraskevi, Greece; koumaras@iit.demokritos.gr; 4 Department of Electronics Technology, University of Malaga, 29016 Malaga, Spain; adz@uma.es; 5 Intel Deutschland GmbH, Lilienthalstraße 4, 85579 Neubiberg, Germany; valerio.frascolla@intel.com

## 1. Introduction

After first being standardized by the 3rd Generation Partnership Project (3GPP) in Release 15, 5th Generation (5G) mobile systems have been rapidly deployed worldwide [1]. This initial phase is being complemented by the release of enhanced versions of the standards, which are expected to enable the evolution of current 5G deployments towards Beyond-5G (B5G) and 6th Generation (6G) systems [2]. Therefore, the need for testing and validating current and planned 5G/B5G technologies and features via data-driven and measurement-based analyses has significantly increased in the last few years, ultimately resulting in key experimental research activities that bring benefit to the entire telecommunications ecosystem, including research institutions, standardization bodies, network operators, technology and equipment providers, enterprises, and verticals [3,4,5,6,7].

On the one hand, experimental studies make it possible to identify how network deployment, configurations, and features affect achievable performance, while guiding the derivation of solutions for system enhancement and optimization. On the other hand, they also bring complex challenges to address, including (but not limited to) the definition of Key Performance Indicators (KPIs) and of proper measurement and testing methodologies. Furthermore, the popular approach of using Artificial Intelligence (AI) and Machine Learning (ML) to address several network aspects clearly requires system experimentation activities in order to be able to properly design AI/ML solutions on top of trustable data-driven analyses [8,9].

Within the above context, the Special Issue “Experimentation in 5G and beyond Networks: State of the Art and the Way Forward” collected original contributions on experimental aspects related to 5G/B5G networks and systems. The call for papers for this Special Issue included topics such as the design, implementation, and usage of 5G/B5G experimental testbeds and platforms, open-source tools for system monitoring and management, end-to-end measurement and validation of 5G/B5G KPIs, and ML/AI-based solutions for the enhancement of different 5G/B5G system aspects, including (but not limited to) New Radio (NR) [10,11], Core Network (CN) [12], Software-Defined Networking (SDN) and Network Function Virtualization (NFV) [13], cloud/edge computing and network slicing [14], and vertical-specific solutions in the context of enhanced Mobile Broadband (eMBB), Ultra-Reliable Low Latency Communication (URLLC) and massive Machine-Type Communication (mMTC) [15].

## 2. Overview of Published Papers

All submissions were judged by their technical merit and relevance, and ten high-quality papers were finally accepted to appear in this Special Issue.

In the following, we provide the list of accepted contributions, followed by a short overview of each contribution after grouping them into three main research areas, i.e., (a) 5G testing and validation, (b) 5G enhancements, and (c) the use of AI/ML for B5G Systems. We believe that such papers will provide key updates and insights into these research areas, and thus may inspire future work aimed towards the further improvement and optimization of next-generation mobile systems.

**List of Contributions**

1. A. Díaz Zayas, G. Caso, Ö. Alay, P. Merino, A. Brunstrom, D. Tsolkas, and H. Koumaras. A Modular Experimentation Methodology for 5G Deployments: The 5GENESIS Approach. Sensors, 20(22):6652, 2020.
2. V. Sanchez-Aguero, I. Vidal, F. Valera, B. Nogales, L. L. Mendes, W. Damascena Dias, and A. Carvalho Ferreira. Deploying an NFV-based Experimentation Scenario for 5G Solutions in Underserved Areas. Sensors, 21(5):1897, 2021.
3. A. Fernández-Fernández, C. Colman-Meixner, L. Ochoa-Aday, A. Betzler, H. Khalili, M. S. Siddiqui, G. Carrozzo, S. Figuerola, R. Nejabati, and D. Simeonidou. Validating a 5G-Enabled Neutral Host Framework in City-Wide Deployments. Sensors, 21(23):8103, 2021.
4. K. Kiela, M. Jurgo, V. Macaitis, and R. Navickas. 5G Standalone and 4G Multi-Carrier Network-in-a-Box Using a Software Defined Radio Framework. Sensors, 21(16):5653, 2021.
5. W. de Oliveira, J. O. R. Batista Jr, T. Novais, S. T. Takashima, L. R. Stange, M. Martucci Jr, C. E. Cugnasca, and G. Bressan. OpenCare5G: O-RAN in Private Network for Digital Health Applications. Sensors, 23(2):1047, 2023.
6. Y. Tian, Y. Bai, and D. Liu. Low-Latency QC-LDPC Encoder Design for 5G NR. Sensors, 21(18):6266, 2021.
7. H. A. Kholidy. Multi-layer Attack Graph Analysis in the 5G Edge Network Using a Dynamic Hexagonal Fuzzy Method. Sensors, 22(1):9, 2021.
8. Y. Z. Bekele and Y.-J. Choi. Random Access Using Deep Reinforcement Learning in Dense Mobile Networks. Sensors, 21(9):3210, 2021.
9. D. G. Riviello, R. Tuninato, E. Zimaglia, R. Fantini, and R. Garello. Implementation of Deep-Learning-based CSI Feedback Reporting on 5G NR-Compliant Link-Level Simulator. Sensors, 23(2):910, 2023.
10. L. Tsipi, M. Karavolos, P. S. Bithas, and D. Vouyioukas. Machine Learning-based Methods for Enhancement of UAV-NOMA and D2D Cooperative Networks. Sensors, 23(6):3014, 2023.

### 2.1. 5G Testing and Validation

Five papers belong to this research area, with the main goal of experimentally analyzing 5G systems and technologies, often in the context of collaborative international projects.

In Contribution 1, Díaz et al. introduce the experimentation methodology defined by the EU H2020 project 5GENESIS, which aims to enable the testing of 5G network components as well as the validation of end-to-end KPIs, e.g., throughput, latency, and service reliability. The proposed methodology is modular, flexible, and open-source, thus enabling its adoption in any 5G testbed. The work also demonstrates the use of the methodology via real-world experiments executed in the 5G Non-Standalone (NSA) [16] network at the University of Malaga.

Arguing about the low effort of researchers and companies in providing Internet access in remote areas, Contribution 2 by Sanchez-Aguero et al. presents the results of 5G-RANGE, an EU H2020 collaborative project between Brazil and the EU. The developed experimentation platform includes a fixed Radio Access Network (RAN) and a network of Unmanned Aerial Vehicles (UAVs). This latter can be dynamically instantiated thanks to the NFV paradigm [13], which supports sporadic communication beyond the boundaries of the fixed RAN. This work presents a preliminary deployment validation by testing voice and data services.

In Contribution 3, Fernández-Fernández at al. present the main goals and achievements of the EU H2020 project 5GCity. This project targets the design and implementation of neutral host solutions, which mobile network operators can use to accommodate the requirements of emerging services by dynamically sharing the same physical infrastructure via network slicing [14]. The proposed 5G-enabled neutral host framework is validated in the cities of Barcelona, Bristol, and Lucca.

In Contribution 4, Kiela et al. present the design and implementation of a Software Defined Radio (SDR)-based Remote Radio Head (RRH) framework. This framework enables researchers to access non-simulated cellular networks more easily, thus reducing system development time and providing test and measurement capabilities for various wireless technologies. The performance of the proposed framework is tested by creating both 4G and 5G Standalone (SA) [16] network-in-a-box, capable of operating in multi-band multi-carrier configurations. Measurement results with a single user show the achievable performance of both networks in terms of downlink/uplink throughput and latency. Moreover, a lower CPU load of the 5G RAN compared to the 4G RAN, and, conversely, a higher CPU load of the 5G Core compared to the 4G Core are observed.

Finally, Contribution 5 by de Oliveira et al. focuses on digital health verticals and presents the efforts of the Brazilian OpenCare5G project to test how 5G technologies can enable timely and efficient digital health services in remote areas. This project uses a 5G private network deployed at the Faculty of Medicine of the University of São Paulo, embedded with an Open RAN (O-RAN) infrastructure [17]. In particular, the work describes the results obtained during the first phase of the project, i.e., the execution of health examinations with portable equipment at different locations within the Faculty of Medicine of the University of São Paulo. A second phase of the project is also expected, with the execution of health exams in remote areas, where onsite personnel is supported by personnel at the University of São Paulo thanks to 5G-enabled efficient communications.

### 2.2. 5G Enhancements

Two papers belong to this research area, with the primary goal of proposing enhancements for current 5G systems.

Contribution 6 by Tian et al. focuses on channel coding for 5G [18]. Considering that Low-Density Parity-Check (LPDC) codes are the 3GPP-standardized coding scheme for 5G NR data channels, this work discusses the design of a Quasi-Cyclic low-latency LPDC (QC-LPDC) scheme, which significantly reduces encoding complexity and resource utilization. By leveraging existing studies, the work showcases a new power-efficient QC-LPDC design, compatible with 5G NR standards and achieving significantly lower encoding latency thanks to a multi-channel parallel coding structure.

Contribution 7 by Kholidy analyzes 5G cybersecurity and discusses security concerns for both 5G networks and connected devices [19]. A method aimed at accurately analyzing 5G vulnerabilities is proposed, which combines the Technique for Order of Preference by Similarity to Ideal Solution (TOPSIS) and hexagonal fuzzy numbers theory. Such a method finds the network graph where attacks might propagate and quantifies both attack costs and network security levels. This work validates the method on a 5G testbed, using a classical TOPSIS-based approach and a vulnerability scanner tool called Nessus as benchmarks [20].

### 2.3. Use of AI/ML for B5G Systems

Three papers belong to this research area, with the main goal of proposing the use of AI/ML to address different aspects of B5G systems.

In Contribution 8 by Bekele et al., the focus is on the random access procedure [21], which end devices execute to connect to the Transmission and Reception Points (TRxPs) forming the cellular RAN. Considering that 5G and B5G systems will increasingly operate in high-frequency spectrum bands, a dense RAN comprising numerous TRxPs is needed to overcome coverage shrinking. This, in turn, increases the challenge for end devices to efficiently select a TRxP in a given time during the random access. To solve this problem, the work casts the random access procedure into an optimization problem and solves it via reinforcement learning. The proposed scheme estimates the congestion of TRxPs and selects an optimal TRxP for each end device during the random access. Through simulation, it is demonstrated that the proposed algorithm improves random access performance by decreasing the average access delay compared to the standard procedure, while also increasing the probability of successful access for end devices.

Contribution 9 by Riviello et al. proposes applying deep learning for the reporting of Channel State Information (CSI) feedback, which is a key aspect in the Frequency Division Duplexing (FDD) 5G and B5G systems [22], where the knowledge of channel characteristics is key to exploiting the full potential of Multiple Input Multiple Output (MIMO) schemes [23]. Therefore, the work designs a framework adopting a convolutional neural network, called NR-CsiNet, aimed at compressing the channel matrix experienced by the user at the receiver side and then reconstructing it at the transmitter side. NR-CsiNet is based on a 5G NR fully compliant simulator that implements a channel generator based on the latest 3GPP channel model. Simulations carried under realistic scenarios, including multi-receiving antenna schemes and noisy downlink channel estimation, show promising results compared to current 5G feedback reporting schemes, in terms of both block error rate and achievable data rate.

Finally, Contribution 10 by Tsipi et al. analyzes a cooperative network including UAVs and device-to-device (D2D) communications, which are expected to play an important role in B5G/6G scenarios [24,25]. In particular, a UAV placement scheme combining supervised and unsupervised ML techniques is proposed to enhance the performance of such a network, where Non-Orthogonal Multiple Access (NOMA) is used as the underlying access scheme. Comparisons against a conventional Orthogonal Multiple Access (OMA) scheme and an alternative scheme using unsupervised ML only show that the proposed approach leads to significant gains in terms of sum rate and spectral efficiency under a varying bandwidth allocation.

## 3. Conclusions

In this Special Issue, we selected ten papers that address different topics related to 5G/B5G experimentation in order to delineate the state of the art and the way forward for further activities in this broad, growing, and important research topic. We hope that the selected articles may provide useful insights into the research areas of 5G testing and validation, 5G enhancements, and use of AI/ML for B5G systems, inspiring future work in the context of these interesting research fields.

We would like to thank all the authors who contributed their work to this Special Issue, as well as the reviewers of the submitted papers who dedicated their time and expertise to providing high-quality reviews that allowed us to finalize a successful Special Issue.
